# Supplementary material for: Team-family conflicts over end-of-life decisions in ICU: A survey of French physicians’ beliefs
Source: PLoS One. 2023 Apr 25;18(4):e0284756. doi: 10.1371/journal.pone.0284756 (PMC10128920; doi:10.1371/journal.pone.0284756)
Supplement: S1 Table — (DOCX) [file pone.0284756.s001.docx]

**English translation of the study questionnaire**

**Team-family conflicts during decision to withhold or withdraw life-sustaining therapies in critically ill patients in the intensive care unit**

The aim of this study is to perform a survey of practices regarding decision-making procedures, and the implementation of decisions to withhold or withdraw life-sustaining therapies in critically ill adult patients in the intensive unit, particularly when there is conflict between the caregiving team and the patient’s family.

In this questionnaire, the term “Family” is intended to mean the members of the patient’s close entourage and/or any surrogate(s). We have deliberately avoided giving a strict definition of what constitutes “conflict”, so that you can report and describe any situations that you personally perceived as conflictual.

*This questionnaire has 20 questions, some have single answers, some are multiple choice questions. There is a free-text box after each question for any comments you may wish to add.*

**1) Among all the patients admitted to your ICU, how often is there an enquiry to find out whether the patient had advance directives?**

***(Please tick one answer only)***

􀀀 Never

􀀀 Rarely

􀀀 Often

􀀀 Very regularly

􀀀 Systematically

Comments :

**2) In your ICU, how often do you estimate that a patient’s written advance directives are made known to you?**

***(Please tick one answer only)***

􀀀 Never

􀀀 Rarely

􀀀 Often

􀀀 Very regularly

􀀀 Systematically

Comments :

**3) When you are planning to have a discussion about withholding or withdrawing life-sustaining therapies, at what point do you ask the patient’s family for their opinion?**

***(Please tick one answer only)***

􀀀 Systematically before any initiatives to withhold or withdraw therapy (WWT) are initiated

􀀀 Before the collegial decision-making meeting, when the medical team is considering WWT

􀀀 After the collegial decision-making meeting, when the decision to WWT has been made

􀀀 Never

Comments :

**4) In your opinion, how often does the family have an opinion that differs from the wishes expressed by the patient (either in advance directives or in any other form)?**

***(Please tick one answer only)***

􀀀 Never

􀀀 Rarely

􀀀 Sometimes

􀀀 Often

􀀀 Always

Comments :

**5) During discussions about WWT, do you agree that requests by the family to pursue treatments that the medical team deems unreasonable pose a problem for the caregiving staff (medical and paramedical)?**

***(Please tick one answer only)***

􀀀 Strongly agree

􀀀 Agree

􀀀 Neither agree nor disagree

􀀀 Disagree

􀀀 Strongly disagree

Comments :

**6) In your opinion, how often are there team-family conflicts further to WWT decisions in your ICU?**

***(Please tick one answer only)***

􀀀 Never

􀀀 Very rarely

􀀀 Now and again

􀀀 Frequently

􀀀 Very frequently

Comments :

**What is the estimated number of such conflicts per year :**

Comments :

**7) Among all the team-family conflicts you have experienced in your career, how often do you think that the WWT decisions were the motive for the conflict?**

***(Please tick one answer only)***

􀀀 Never

􀀀 In a few cases

􀀀 In about half of all cases

􀀀 In the majority of cases

􀀀 Always

Comments :

**8) In your ICU, in most cases, when does the conflict about WWT decisions become apparent?**

***(Please tick one answer only)***

􀀀 Prior to admission to ICU (when a decision not to admit to ICU is made, or when a decision is made to admit to ICU but with limited therapeutic engagement)

􀀀 Before the collegial decision-making meeting is held

􀀀 Before the collegial decision-making meeting is held

􀀀 When the WWT decision is going to be implemented (definitive extubation, for example)

Comments:

**9) In your ICU, how does conflict about WWT decisions become apparent?**

***(You may tick more than one answer)***

􀀀 Discussions between the family and paramedical staff

􀀀 Discussions between the family and a junior doctor (resident)

􀀀 Discussions between the family and a senior doctor

􀀀 Letter to the hospital management

􀀀 Letter to the Chief of the ICU

􀀀 Aggressive attitude and/or physical or verbal threats to caregivers

􀀀 Request to transfer the patient to another unit

􀀀 Alert given by an outside doctor (e.g. the general practitioner)

􀀀 Other : Please specify :…………………………………………

Comments:

**10) Usually, the family are opposed to ….**

***(You may tick more than one answer)***

􀀀 Discontinuation of treatment.

􀀀 Limitation of treatment (non-initiation, or non-optimisation)

􀀀 The terms of implementation of a WWT decision

􀀀 Other : Please specify :…………………………………………

Comments:

**11) Based on what families tell you or reproach you with, what factors do you think are responsible for team-family conflicts surrounding WWT decisions?**

***(Never, Rarely, Sometimes, Most of the time, All the time)***

1. Factors related to medical practice

􀀀 Speed of the decision to WWT in relation to the admission to ICU

􀀀 On the contrary, a prolonged stay in ICU

􀀀 Absence of a reference senior physician for the patient / multiple doctors caring for patient

􀀀 Decision to WWT taken prematurely, when the clinical situation is stable

􀀀 Lack of communication on the part of the caregiving staff

2. Factors related to the context

􀀀 Lack of advance directives

􀀀 Lack of a designated surrogate

􀀀 Ethnic, cultural or religious issues

􀀀 Conflicts within the family

􀀀 Too many persons in the patient’s entourage

3. Factors related to the family

􀀀 Denial of the situation (persisting hope of recovery or improvement)

􀀀 Semantic association equating WWT with death

􀀀 Uncertainty about the prognosis/ acceptance of substantial handicap

􀀀 Fear that the patient will suffer or be uncomfortable

􀀀 Fear that the patient is being abandoned by the caregiving staff

􀀀 General deterioration of the relationship between the medical staff and the family

Comments:

**Management of conflict**

**12) What tool(s) do you use to try to resolve team-family conflicts surrounding WWT decisions?**

***(Never, Rarely, Sometimes, Most of the time, All the time)***

􀀀 Regular interviews with the family

􀀀 Offer psychological support for the family

􀀀 Call on a religious representative

􀀀 Call on the hospital’s mediator

􀀀 Mobile palliative care team

􀀀 Ask the treating physician (general practitioner) to get involved

􀀀 Get an opinion from another physician outside the ICU

􀀀 Transfer the patient to another ICU

􀀀 Involve the local ethics review committee

􀀀 Ask for a clinical ethics consultation

􀀀 Call on the legal department

􀀀 The Department Chief manages the conflict

􀀀 Conflict is managed internally, without outside help

􀀀 Allow some time to elapse

Comments:

**13) Which of these solutions have proven to be useful? Which are inefficacious? Why?**

**14) In your opinion, how often are team-family conflicts surrounding WWT decisions resolved prior to the patient’s discharge or death?**

***(Please tick one answer only)***

􀀀 Never

􀀀 Rarely

􀀀 Sometimes

􀀀 Often

􀀀 Always

Comments:

**15) Usually, when the conflict is resolved, it is because …..**

***(Please tick one answer only)***

􀀀 The family have finally accepted the WWT decision (possibly with the help of the solutions mentioned in question 12)

􀀀 You suspended the WWT decision

􀀀 The patient died before the WWT decision could be implemented

􀀀 The patient died after implementation of the WWT decision and the family feels a form of “relief”

􀀀 Other : please specify : ………………………………………………………….

Comments:

**Impact of conflict**

**16) In your opinion, what are the possible consequences of team-family conflict on the caregiving staff (medical and paramedical)? (*Strongly agree, Agree, Neither agree nor disagree, Disagree, Strongly disagree*)**

􀀀 A feeling that the principles of beneficence and non-maleficence towards the patient were not respected

􀀀 Burnout

􀀀 Infringement on their right to exercise their profession

􀀀 Fear of legal repercussions

􀀀 It could damage team cohesion

􀀀 Infringement of the principle of distributive justice – feeling in inequality in access to medical resources

􀀀 Patient management could lose its meaning

􀀀 Staff could lose their motivation to work

􀀀 Creates anxiety for the team on night-duty

􀀀 Feeling that management has failed

􀀀 Feelings of guilt

Comments:

**17) In your opinion, what are the possible consequences of team-family conflict on your practice? (*Strongly agree, Agree, Neither agree nor disagree, Disagree, Strongly disagree*)**

􀀀 Do rounds less often – avoidance behaviours

􀀀 Give fewer explanations (to the patient or the family)

􀀀 Less psychological support (for the patient or the family)

􀀀 Increase paraclinical examinations

􀀀 Decrease paraclinical examinations

􀀀 Allow only senior doctors to deal with the case – exclude students

􀀀 Require change or turnover in caregiving team

􀀀 Increase time dedicated to the patient – impact on time dedicated to other patients

􀀀 Improve the note-taking and recording in the patient’s file and traceability

Comments:

**18) Do you think that when team-family conflict exists, the WWT decision-making process is better respected, as regards the legislation?**

***(Please tick one answer only)***

􀀀 Yes

􀀀 No

Comments :

**19) Usually, when there is conflict surrounding a WWT decision, you …..**

***(You may tick more than one answer)***

􀀀 Suspend the decision

􀀀 Re-evaluate the decision, in a new ethics meeting

􀀀 Apply the decision progressively

􀀀 Apply the decision as you usually would if there was no conflict

Comments:

**20) In your opinion, which of the following propositions could help to avoid team-family conflicts surrounding WWT decisions?**

**(*Strongly agree, Agree, Neither agree nor disagree, Disagree, Strongly disagree*)**

􀀀 Systematically enquire (whenever possible) about the existence of advance directives for every patient admitted to the ICU

􀀀 Allow free visiting hours to facilitate the presence of the family at the patient’s bedside

􀀀 Invite the family to participate in the care of the patient

􀀀 Involve the family in decisions right from the beginning of management

􀀀 Involve the family in the collegial decision-making process

􀀀 Systematic presence of a psychologist when WWT decisions are being announced to the family

􀀀 Hold formal family interviews, in a dedicated room, at a dedicated time

􀀀 Involve the palliative care team from an early stage

􀀀 Distribute a “family information booklet” dedicated to WWT and comfort care, whenever WWT decisions are being considered

􀀀 Create a standardized protocol for WWT in each unit

􀀀 When WWT is being considered, emphasize how burdensome ICU therapy is.

􀀀 Other: please specify:…………………………………………………

Comments :
