# Supplementary figures and images for: Team-family conflicts over end-of-life decisions in ICU: A survey of French physicians’ beliefs
Source: PLoS One. 2023 Apr 25;18(4):e0284756. doi: 10.1371/journal.pone.0284756 (PMC10128920; doi:10.1371/journal.pone.0284756)

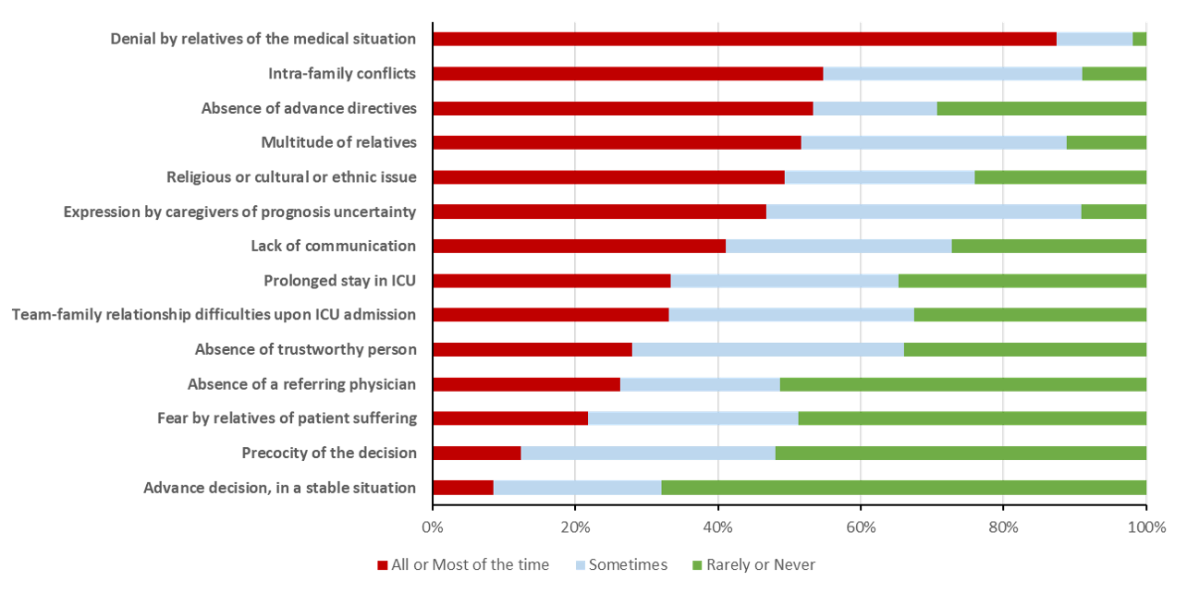

Supplement: S1 Fig — (TIF) [file pone.0284756.s002.tif]

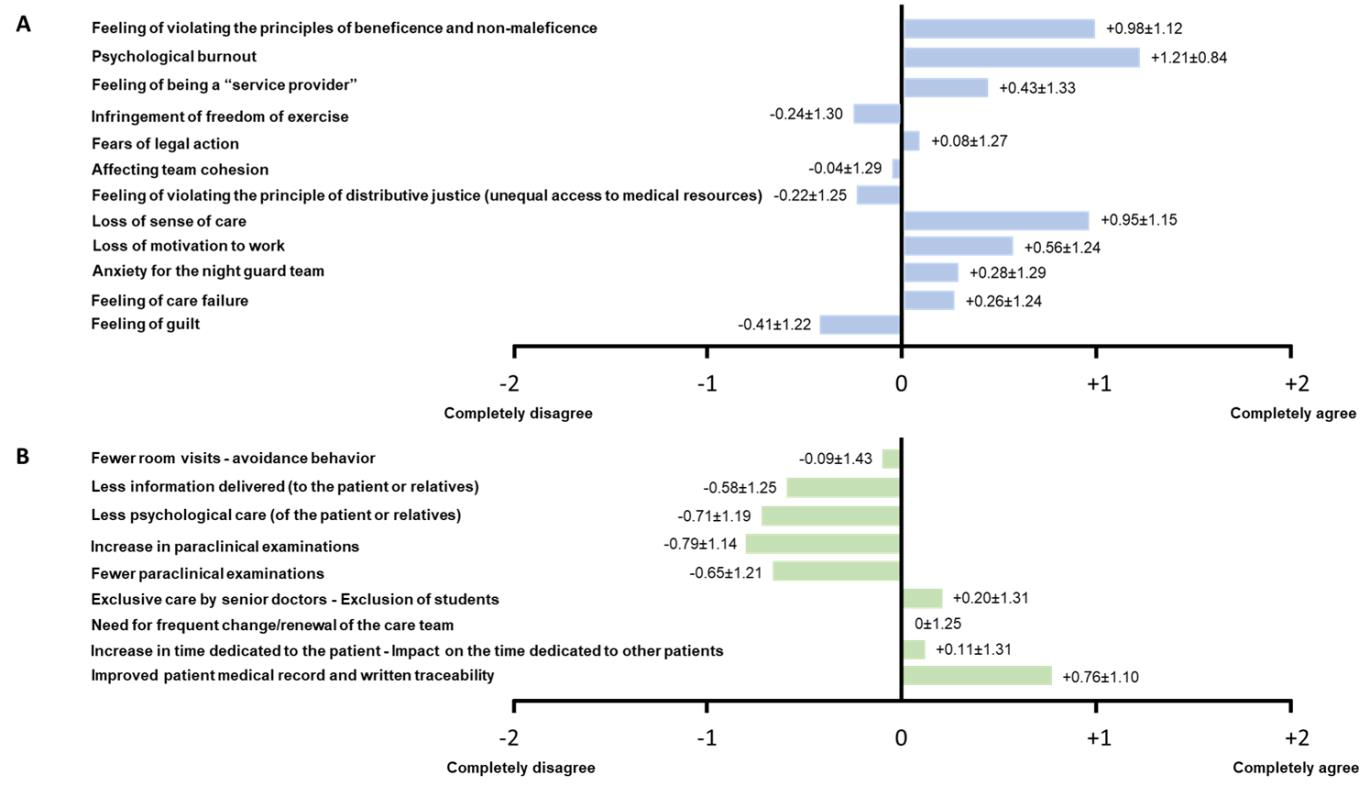

Supplement: S2 Fig — A. On caregivers; B. On patient care. Data are expressed as mean (±SD) on a Likert scale ranging from “completely disagree” (-2) to “completely agree” (+2). (TIF) [file pone.0284756.s003.tif]
