# Supplementary material for: Team-family conflicts over end-of-life decisions in ICU: A survey of French physicians’ beliefs
Source: PLoS One. 2023 Apr 25;18(4):e0284756. doi: 10.1371/journal.pone.0284756 (PMC10128920; doi:10.1371/journal.pone.0284756)
Supplement: S1 File — (DOCX) [file pone.0284756.s004.docx]

**Summary of the French LST limitation decision-making process**

For any LST decision, French law requires that a collegial procedure be followed.

Situations requiring a collegial procedure:

- LST limitation decision as a refusal of unreasonable obstinacy when the patient is unable to express his/her wishes.
- Verification of the conditions for the implementation of deep and continuous sedation, at the patient’s request or following a LST limitation decision.
- Verification of the conditions for non-application of advance directives when they appear manifestly inappropriate or inconsistent with the situation.

Elements of the collegial procedure defined by regulation:

- Collection of the patient’s wishes: search for advance directives and, in their absence, testimony from the trustworthy person or, failing this, of the family or close friends.
- Informing of the trustworthy person or, failing that, the family or close friends of the holding of a collegial procedure and the reasons for it.
- Opinion of a doctor called as a consultant without any hierarchical link with the doctor in charge of the patient. The consultant refers to a physician who has the knowledge, experience, perspective and impartiality to assess the situation as a whole. He or she is not necessarily a specialist or an expert in the matter (the technical opinions of expert’s advice is given before the decision is made).
- Consultation with the medical and non-medical caregivers’ teams.
- Reasoned decision made by the physician in charge of the patient.
- Recording of the reasons for the decision in the medical record.
- Informing the trustworthy person or, failing that, the family or close friends of the nature of and reasons for the decision.
- Notification of the decision to the persons from whom the physician has inquired about the patient’s wishes.

Legal recourse in case of disagreement of relatives with the decision:

In case of disagreement, the law allows family members to object to the decision. Appeals against LST limitation decisions are subject to a procedure known in French law as “référé-liberté” and are made to the Administrative Court. These decisions of the administrative court can themselves be appealed to the Council of State (“Conseil d'Etat”), the highest French jurisdiction.

References:

- Quenot JP, Ecarnot F, Meunier-Beillard N, Dargent A, Large A, Andreu P, et al. What are the ethical aspects surrounding the collegial decisional process in limiting and withdrawing treatment in intensive care? Ann Transl Med. 2017 Dec;5(Suppl 4):S43
- M. Le Dorze, F. Claudot, P.F. Perrigault et al., Judiciarisation de la fin de vie en réanimation : quand les proches demandent la poursuite des traitements, Éthique et santé, https://doi.org/10.1016/j.etiqe.2022.03.001
- Law 2002-303 dated 4 March 2002 regarding patients rights and the quality of the healthcare system. Journal Officiel de la Republique Francaise; 4 March 2002. Available online: https://www.legifrance.gouv.fr/ affichTexte.do?cidTexte=JORFTEXT000000227015&cate gorieLien=id
- Law 2005-370 dated 22 April 2005 regarding patients rights and end-of-life. Journal Officiel de la Republique Française; 23 April 2005 - Légifrance [Internet]. Available from: <https://www.legifrance.gouv.fr/jorf/id/JORFTEXT000000446240/>
- Law 2016-87 dated 2 February 2016 introducing new rights for patients and persons at the end-of-life. Journal Officiel de la République Française; 3 Februray 2016 - Légifrance [Internet]. Available from: <https://www.legifrance.gouv.fr/jorf/id/JORFTEXT000031970253/>
- Décision n° 2017-632 QPC du 2 juin 2017 | Conseil constitutionnel [Internet]. [cited 2023 Feb 22]. Available from: https://www.conseil-constitutionnel.fr/decision/2017/2017632QPC.htm
