# Supplementary material for: Team-family conflicts over end-of-life decisions in ICU: A survey of French physicians’ beliefs
Source: PLoS One. 2023 Apr 25;18(4):e0284756. doi: 10.1371/journal.pone.0284756 (PMC10128920; doi:10.1371/journal.pone.0284756)
Supplement: S2 File — (DOCX) [file pone.0284756.s005.docx]

**Knowledge of the patient’s wishes**

Concerning the expression of the patient’s wishes regarding his/her EOL conditions, 39% of the responding physicians declared that they systematically look for written ADs for all patients, but only 9% believe that ADs are frequently brought to their attention.

When the opinion of relatives is sought regarding possible LST limitation, it is done so before any LST limitation process by 31% of the physicians, before the collegial meeting for 51% of the physicians, and after the collegial meeting for 18% of the physicians. About half of responding physicians (54%) believe that the opinion expressed by the relatives may be the relatives’ own opinion, rather than an accurate expression of the patient’s wishes.
